# Scleritis in Iran

**DOI:** 10.1186/s12348-025-00473-x

**Published:** 2025-03-05

**Authors:** Sahba Fekri, Reza Esmaili Fallah, Masoud Soheilian, Seyed-Hossein Abtahi, Hosein Nouri

**Affiliations:** 1 Ophthalmic Research Center, Research Institute for Ophthalmology and Vision Science, Shahid Beheshti University of Medical Sciences, Tehran, Iran; 2 Department of Ophthalmology, Labbafinejad Medical Center, Shahid Beheshti University of Medical Sciences, Tehran, Iran; 3 School of Medicine, Isfahan University of Medical Sciences, Isfahan, Iran

**Keywords:** Scleritis, Scleral disease, Autoimmune disease, Immunosuppressants, Uveitis

## Abstract

**Purpose:**

To present the demographic and clinical presentations of anterior scleritis among Iranian patients.

**Methods:**

This retrospective case-series at a tertiary center in Iran, identified and analyzed anterior scleritis cases admitted from 2008 to 2018. Extracted data included demographics, clinical features, background systemic diseases, utilized therapies, and follow-up data on visual outcomes, ocular complications, and recurrence rate. Patients with incomplete records were excluded from the analysis.

**Results:**

Sixty-five patients (83 eyes) with anterior scleritis were included, with a female predilection (77%) and a mean age (± SD; range) of 44.8 (± 14.6; 11–81). Diffuse and necrotizing scleritis were the most and least common subtypes, respectively. Bilateral involvement (28% at baseline, 44.6% eventually) and concurrent keratitis (10.7%) or uveitis (16.9%) were documented in some cases. Most cases were idiopathic (61.5%). Scleritis was the initial manifestation of autoimmune diseases in six patients. In addition to oral and/or intravenous corticosteroid therapy, most of our patients (70.7%) were treated with immunosuppressive regimens. No inter-subtype difference was noted in recurrence rate and time to treatment cessation. Necrotizing subtype was associated with worse visual outcomes and more frequent ocular complications.

**Conclusion:**

Despite limitations in data collection and follow-up, our findings contribute valuable insights into the clinical characteristics and management of scleritis in a group of Iranian patients for the first time.

## Background

Scleritis, an infrequent ocular inflammatory disorder involving the sclera and potentially the surrounding tissues, predominantly affects middle-aged individuals, with a predilection for females [1–3]. Incidence rates, as estimated by population-based studies, range from 2.8 to 4.2 per 100,000 person-years [2, 4, 5].

Patients often present with gradual-onset ocular pain, redness, photophobia, or visual decline. The specific symptoms depend on the subtype of scleritis [6]. The prevailing classification system takes the anatomical location (anterior or posterior) and clinical appearance of scleral inflammation (diffuse, nodular, or necrotizing) into account [7]. Scleritis may vary in severity from benign inflammation to scleral necrosis. Severe forms, recurrences, and inadequate care confer risk for vision loss [6].

Approximately, one-third of individuals presenting with scleritis have a documented systemic autoimmune disorder, such as rheumatoid arthritis (RA), granulomatosis with polyangiitis (GPA), and systemic lupus erythematosus (SLE) [2, 6, 8]. Notably, scleritis may surface as the first manifestation of systemic autoimmune diseases in around 9% of patients [2]. If present, such concomitant diseases necessitate intensified therapeutic regimens, and thus, should be actively screened [6].

The rarity of scleritis has precluded the study of its demographic and clinical characteristics across a sizeable number of patients in Iran. Therefore, Iranian patients are under-represented, if represented at all, in the existing literature. Aimed at addressing this gap, we retrospectively analyzed a relatively large series of scleritis cases referred to a tertiary eye care center in Tehran, Iran, spanning from 2008 to 2018.

## Methods

This retrospective case-series analyzed the records of patients diagnosed with scleritis at the Uveitis Clinic of Labbafinejad Medical Center, a tertiary academic center affiliated with Shahid Beheshti University of Medical Sciences, Tehran, Iran, between March 20, 2008 and March 21, 2018. We conducted a search of hospital records using the respective International Classification of Diseases (ICD) codes (9th and 10th revisions), i.e., 379.0 (ICD-9) and H15.0 (ICD-10; effective from 2016). Full records of identified anterior scleritis cases were then retrieved for data extraction.

The diagnosis of anterior scleritis was made by uveitis specialists based on clinical findings, thorough ophthalmic examinations, and confirmatory application of topical phenylephrine 10%. Scleritis subtype was determined using Watson and Hayreh’s classification system [7]. We excluded patients with presumed posterior scleritis due to the lack of clear diagnostic approaches, work-up, and documentation.

We extracted the following data from patients’ documents after anonymization: demographics, presenting complaints, history of systemic diseases (including chronic infections and autoimmune disorders), scleritis subtype and laterality, concurrent involvement of cornea or uvea, instituted therapeutic agents for acute management and maintenance, best-corrected visual acuity (BCVA) in the affected eye(s) at baseline and the last follow-up visit, recurrence or ocular complications during follow-up, time of scleritis resolution and treatment discontinuation, and results from laboratory investigations and systemic work-up in collaboration with the rheumatology service at the center.

Routine laboratory investigations included complete blood count with differential, chest radiography, erythrocyte sedimentation rate (ESR), c-reactive protein (CRP), liver function tests, angiotensin converting enzyme (ACE), rheumatoid factor, cytoplasmic and perinuclear anti-neutrophil cytoplasmic autoantibody (c-ANCA and p-ANCA, respectively), antinuclear antibody, anti-cyclic citrullinated peptide (anti-CCP) antibody, Venereal Disease Research Laboratory test, Fluorescent treponemal antibody absorption (FTA-Abs) test, purified protein derivative (PPD) skin test, human immunodeficiency virus (HIV) 1/2 antibodies, hepatitis C virus (HCV) antibody, hepatitis B surface antigen (HBs Ag), Toxoplasma gondii antibody, human T-cell lymphoma virus-1 (HTLV-1) antibody, and Human leukocyte antigen (HLA) serotyping (for HLA-B27, HLA-B5, and HLA-B51).

This study adhered to the principles of the Declaration of Helsinki. Participants’ data were anonymized before extraction. The study protocol was approved by the institutional review board (IR.SBMU.MSP.REC.1398.174), and a waiver for obtaining patients’ consent was granted due to the retrospective nature of the study, provided that individuals’ anonymity was observed at all stages of the study.

We reported descriptive statistics using frequency, percentage, mean (and standard deviation [SD]), or median (and interquartile range [IQR]), depending on the variable type and normality of distribution. We used relevant grouping variables, such as scleritis subtype, to enable inter-group comparisons, employing independent samples t-test, Mann–Whitney U test, chi-square test, ANOVA, or Kruskal–Wallis test, depending on the grouping factor and variable type or distribution. Decimal BCVA values were converted to Logarithm of the Minimum Angle of Resolution (LogMAR), and baseline and final values were compared using the Wilcoxon signed-rank test. A *p*-value < 0.05 was considered statistically significant. We used IBM SPSS Statistics for Windows, version 26 (IBM Corp., Armonk, N.Y., USA).

## Results

In this retrospective case-series, 65 patients (83 eyes) with anterior scleritis were included. A summary of their demographic information, clinical characteristics, and treatment modalities is shown in Table 1. There were 15 male patients (23%) and 50 female patients (77%), with similar mean ages (45.3 ± 12.1 vs. 42.8 ± 14.6, respectively, *p* = 0.55), ranging from 11 to 81 years. Eighteen patients (28%) had bilateral scleritis at presentation. Concurrent keratitis was documented in 11% of patients (*n* = 7), and anterior uveitis in 17% (*n* = 11).

**Table 1:** Demographic and clinical features of patients (*n* = 65)

Variable	Frequency/mean
**Age,** mean ± SD (Range) (years)	44.85 ± 14.62 (11–81)
**Sex**	
Male	15 (23%)
Female	50 (77%)
**Laterality**	
Unilateral	47 (72%)
Bilateral	18 (28%)
**Chief complaints**	
Redness ± ocular pain	52 (80%)
Photosensitivity	10 (15%)
Decreased vision	3 (5%)
**Past medical history**	
Diabetes mellitus	3 (5%)
Systemic hypertension	9 (14%)
Breast cancer	1 (1.5%)
**Anterior scleritis subtype**	
Diffuse	37 (57%)
Nodular	19 (29%)
Necrotizing	9 (14%)
**Etiology**	
Infectious	1 (1.5%)
Autoimmune	24 (36.9%)
Idiopathic	40 (61.5%)
**Anterior segment involvement**	
Anterior uveitis	11 (17%)
Peripheral ulcerative keratitis	7 (11%)
**Treatment**	
Intravenous Methylprednisolone	14 (21.5%)
Oral corticosteroid	65 (100%)
Oral NSAID	7 (11%)
Immunosuppressants	46 (71%)
* Azathioprine*	23 (35%)
* Methotrexate*	10 (15%)
* Mycophenolate mofetil*	3 (5%)
* Cyclosporine*	3 (5%)
* Cyclophosphamide*	7 (11%)

The determined etiology was infectious in one patient, immune-mediated in 24 patients, and idiopathic in the remaining 40 individuals. In 6/24 immune-mediated cases, scleritis was the first manifestation of the disorder. Table 2 presents the prevalence of underlying systemic autoimmune diseases among patients. In the entire series, the most frequent immune-mediated systemic diseases were RA (13.8%), GPA (9.2%), and SLE (4.6%). Patients with necrotizing scleritis had concurrent RA or GPA more frequently (Table 3). One patient with nodular scleritis had high serum angiotensin converting enzyme levels, for whom sarcoidosis was confirmed histopathologically. The serum samples of all patients tested negative for p-ANCA, FTA-Abs, Toxoplasma gondii antibody, HIV-1/2 antibodies, HCV antibody, and HBs Ag. Elevated ESR or CRP levels were not associated with persistence, recurrence, or necrotizing appearance of scleritis (*p* > 0.05).

**Table 2:** Underlying immune-mediated diseases in patients with anterior scleritis (*n* = 65)

Systemic disease	Previously known	Newly diagnosed during screening/follow-up
Total no. (%)	18 (28%)	6 (9%)
Rheumatoid arthritis	9 (14%)	-
Systemic lupus erythematosus	2 (3%)	1 (1.5%)
Granulomatosis with polyangiitis	4 (6.2%)	2 (3%)
Psoriatic arthritis	1 (1.5%)	-
Inflammatory bowel disease	2 (3%)	-
Behcet’s disease	-	2 (3%)
Sarcoidosis	-	1 (1.5%)

**Table 3:** Underlying immune-mediated diseases based on scleritis subtype

	**Type of anterior scleritis**		
**Systemic disease**	**Diffuse,** (*n* = 37)	**Nodular,** (*n* = 19)	**Necrotizing,** (*n* = 9)
Idiopathic	25 (67%)	13 (68%)	3 (33%)
Rheumatoid arthritis	2 (5%)	4 (21%)	3 (33%)
Systemic lupus erythematosus	3 (8%)	-	-
Granulomatosis with polyangiitis	2 (5%)	1 (5%)	3 (33%)
Psoriatic arthritis	1 (3%)	-	-
Inflammatory bowel disease	2 (5%)	-	-
Behcet’s disease	2 (5%)	-	-
Sarcoidosis	-	1 (5%)	-

All of our patients received oral corticosteroids as first-line treatment. The severity of scleritis necessitated intravenous methylprednisolone pulse therapy in ~ 21% of our patients (*n* = 14), and immunosuppressants were initiated in 71% of cases (*n* = 46). Oral non-steroidal anti-inflammatory drugs were started for 7 patients because corticosteroids were tapered earlier (Table 1) – due to poorly controlled diabetes mellitus or steroid-related systemic side effects.

Follow-up data (≥ 3 months) were available for 59 eyes of 47 individuals. Information on recurrence and ocular complications across subtypes are provided in Table 4. No inter-subtype difference was noted regarding the recurrence rate among patients. Eleven patients experienced recurrences in their fellow, previously unaffected eyes (8 in diffuse and 3 in necrotizing subgroups).

**Table 4:** Follow-up data on recurrence and ocular complications

	**Type of anterior scleritis**				
	**Diffuse, **(*n*=38)	**Nodular, **(*n*=12)	**Necrotizing, **(*n*=9)	**Total, **(*n*=59)	***p*-value**
**Recurrence**	12 (31.6%)	3 (25.0%)	3 (33.3%)	18 (30.5%)	0.805
**Ocular complications**					0.023
Cataract	4 (10.5%)	-	1 (11.1%)	5 (8.5%)	
Ocular hypertension	7 (18.4%)	1 (8.3%)	2 (22.2%)	10 (16.9%)	
Ocular hypotony	-	-	1 (11.1%)	1 (1.7%)	
Scleral thinning	3 (7.9%)	1 (8.3%)	8 (88.9%)	12 (20.3%)	
Complete vision loss (no light perception)	1 (4%)	-	1 (11.1%)	2 (3.4%)	
Scleral perforation	-	-	1 (11.1%)	1 (1.7%)	

Median (IQR) BCVA values at baseline and the last follow-up were 0.045 (0.188) and 0.047 (0.150) LogMAR, respectively (*p*-within = 0.32). Eventually, complete vision loss (no light perception) occurred in two patients. Inter-subtype differences in BCVA were not significant at baseline (median [range] in logMAR: 0.05 [0.0 – 0.70] in diffuse, 0.05 [0.0 – 1.30] in nodular, and 0.70 [0.0 – 0.78] in necrotizing subgroups, *p* = 0.063), but were so at the last follow-up (median [IQR] in logMAR: 0.0 [0.0 – 0.70] in diffuse, 0.050 [0.0 – 1.0] in nodular, and 0.52 [0.22 – 0.70] in necrotizing subgroups, *p* = 0.037). The difference between necrotizing and diffuse subtypes was significant (*p* = 0.031). The overall mean ± SD (range) time to treatment discontinuation was 17.96 ± 16.79 months (2–69). Eyes with diffuse, nodular, and necrotizing subtype had comparable mean (SD) time to treatment cessation (18.5 [2.1], 21.7 [13.3], 15.8 [20.6] months, respectively; *p* = 0.177).

## Discussion

We described the characteristics of a relatively large group of Iranian patients with anterior scleritis for the first time. Only three previous studies from Turkey [9], Saudi Arabia [10] and Egypt [11] have described scleritis features in Middle Eastern individuals. An analysis of scleritis cases documented by the American Academy of Ophthalmology Intelligent Research in Sight (IRIS) Registry showed that clinical characteristics of scleritis may vary based on ethnicity and region of residence [3]. Our studied population included Caucasians.

As previously reported [2, 3, 10–20], we observed a striking female predominance (77%; female-to-male ratio ~ 3:1). Other cohorts with over 70% female patients include Turkish (71.9%), Italian (74%) [15], American/Spanish (71%) [14], Puerto Rican (Hispanic, 70.2%) [13], and Egyptian cohorts (74.9%) [11].

The mean age at onset in our cohort was 44.8 years, which falls within the range reported by previous studies (41.5–59.5 years) [2, 3, 9–21]. Our patients tended to be slightly younger than those from most other studies, who were typically affected in their 50’s [3, 10, 12–17, 19, 21]. Only the Bangladeshi (42 years) [20] and Indian (41.5 years) cohorts [19] had younger mean ages than ours.

We only included cases of anterior scleritis. The most common presenting complaint was red eye (80%), usually accompanied by ocular pain. In agreement with earlier reports [12–16, 18–20], the most prevalent subtype of anterior scleritis was diffuse, and the least common was necrotizing. Exceptions in the literature, where nodular scleritis was the most frequent subtype, were cohorts from Turkey (43%) [9], Korea (40.8%) [21], and Egypt (44.9%) [11].

In the present study, both eyes were affected in 44.6% of patients, most of whom (27.7%) had bilateral involvement at baseline. This is consistent with almost all other studies, reporting bilateral involvement in approximately 25–50% of patients [2, 3, 9–16, 18–21].

Autoimmune disorders were present in 36.9% of our patients, with 27.7% diagnosed before the incidence of scleritis and 7.8% diagnosed after that. The most prevalent associated disorders were RA (13.8%) and GPA (9.2%), in line with previous reports [11–16, 18–20]. SLE, IBD, psoriasis, and sarcoidosis were also documented (Tables 2 and 3). Notably, RA was significantly more common in patients with necrotizing scleritis (Table 3).

Overall, systemic autoimmune disorders and infection accounted for 36.9% and 1.5% of our cases, respectively. The remaining 61.5% were classified as idiopathic. This is consistent with the majority of other tertiary care center cohorts, which reported immune-mediated causes in 21–36% of cases [10–16, 18, 19]. However, a few cohorts have found less frequent autoimmune associations, such as the Taiwanese (12.9% immune-mediated; 38.7% infectious, mostly related to pterygium surgery), Indian (8.3% immune-mediated; 30% infectious), and Bangladeshi cohorts (17.7% immune-mediated). Possible explanations for these differences may include insufficient rheumatologic diagnostic work-up [20] or a high number of infectious cases [17, 19].

Referral bias cannot be ruled out for the above-mentioned studies and the present work, as these reports included patients referred to tertiary centers, whose condition might have been more complex and severe. A recent analysis of approximately 5.9 million electronic medical records at Johns Hopkins Medicine Health System demonstrated that: i) 30.8% of scleritis patients had an associated autoimmune disease, ii) RA was the most common immune-mediated cause of scleritis (6.8% of cases), followed by HLA-B27 (5.7%), Sjögren's syndrome (4.5%), SLE (3.0%), GPA (1.7%), and sarcoidosis (1.5%), and iii) GPA carried the highest risk for scleritis (relative risk [RR] = 117) – far greater than RA with a RR of 20.2 [22].

Regarding the visual outcome, necrotizing scleritis was associated with the worst visual acuity both at presentation and the last visit (median BCVA of 0.70 and 0.52 LogMAR, respectively), aligning with results from previous studies [10, 11, 21]. The probability of remaining complication-free was also lowest in this subgroup (Table 4).

Like other retrospective studies, this work has limitations regarding possible biases in data collection and analysis. A considerable proportion of our cases (18/65) were lost to follow-up, resulting in insufficient power to analyze treatment outcomes and their association with background conditions and treatment regimens.

## Conclusion

To summarize, our study showed that among Iranian patients, anterior scleritis affected middle-aged individuals with a female predilection. Hyperemia, typically painful, was the most common clinical presentation. Scleritis was unilateral in most patients, and its prevailing subtype was diffuse anterior. The determined etiology was immune-related in about one-third of patients, but infections were rarely responsible. Background immune-mediated conditions included RA, GPA, SLE, IBD, psoriasis, and sarcoidosis. The subtype of scleritis did not appear to affect recurrence rates. The visual prognosis was favorable in the majority of patients, except for those with necrotizing scleritis.

## Data Availability

No datasets were generated or analysed during the current study.

## Abbreviations

ACE: Angiotensin converting enzyme
anti-CCP: Anti-cyclic citrullinated peptide antibody
BCVA: Best-corrected visual acuity
c-ANCA: Cytoplasmic anti-neutrophil cytoplasmic autoantibody
CRP: C-reactive protein
ESR: Erythrocyte sedimentation rate
FTA-Abs: Fluorescent treponemal antibody absorption
GPA: Granulomatosis with polyangiitis
HBs Ag: Hepatitis B surface antigen
HCV: Hepatitis C virus
HIV: Human immunodeficiency virus
HLA: Human leukocyte antigen
HTLV-1: Human T-cell lymphoma virus-1
IBD: Inflammatory bowel disease
ICD: International Classification of Diseases
IQR: Interquartile range
LogMAR: Logarithm of the minimum angle of resolution
p-ANCA: Perinuclear anti-neutrophil cytoplasmic autoantibody
PPD: Purified protein derivative
RA: Rheumatoid arthritis
RR: Relative risk
SD: Standard deviation
SLE: Systemic lupus erythematosus
